# The spectrum of acute bacterial meningitis in elderly patients

**DOI:** 10.1186/1471-2334-13-108

**Published:** 2013-02-27

**Authors:** Pere Domingo, Virginia Pomar, Natividad de Benito, Pere Coll

**Affiliations:** 1 Infectious Diseases Unit, Hospital de la Santa Creu i Sant Pau, Universitat Autònoma de Barcelona, Av. Sant Antoni Mª Claret, 167, Barcelona 08025, Spain; 2 Department of Microbiology, Hospital de la Santa Creu i Sant Pau, Universitat Autònoma de Barcelona, Barcelona, Spain

**Keywords:** Bacterial meningitis, Acute, Elderly, *Streptococcus pneumoniae*, *Listeria monocytogenes*, Co-morbidities, Outcome, Complications, Post-meningitic sequelae

## Abstract

**Background:**

We conducted a prospective, observational study in Barcelona to determine the epidemiology, clinical features, and outcome of elderly patients with acute bacterial meningitis (ABM) compared with younger adults.

**Methods:**

During 1982–2010, all patients with ABM were prospectively evaluated. There were two groups: I (15–64 years) and II (≥ 65 years). All patients underwent clinical examination on admission and at discharge following a predefined protocol.

**Results:**

We evaluated 635 episodes of ABM. The incidence was 4.03/100,000 (Group I) and 7.40 /100,000 inhabitants/year (Group II) (RR = 1.84; 95%CI: 1.56–2.17, P < 0.0001). Elderly patients had co-morbid conditions more frequently (P < 0.0001) and more frequently lacked fever (P = 0.0625), neck stiffness (P < 0.0001) and skin rash (P < 0.0001), but had an altered level of consciousness more often (P < 0.0001). The interval admission-start of antibiotic therapy was longer for elderly patients (P < 0.0001). Meningococcal meningitis was less frequent in elderly patients (P < 0.0001), whereas listerial (P = 0.0196), gram-negative bacillary (P = 0.0065), and meningitis of unknown origin (P = 0.0076) were more frequent. Elderly patients had a higher number of neurologic (P = 0.0009) and extra-neurologic complications (P < 0.0001). The overall mortality ratio was higher in elderly patients (P < 0.0001).

**Conclusions:**

Elderly people are at higher risk of having ABM than younger adults. ABM in the elderly presents with co-morbid conditions, is clinically subtler, has a longer interval admission-antibiotic therapy, and has non-meningococcal etiology. It is associated with an earlier and higher mortality rate than in younger patients.

## Background

During the past years we have witnessed significant changes in the epidemiology of ABM. The most important change is the dramatic decline of incidence of meningitis due to *Haemophilus influenzae* serotype b (Hib) due to infant vaccination [1,2], and the striking decrease of early-onset disease caused by group B Streptococcus because of use of intra-partum antibiotic prophylaxis [3].

The incidence and pattern of many infectious diseases vary over time. These changes are due to many factors, but among them modifications in the population structure stand out. Population structure in developed countries is continuously changing, and this change is mainly driven by two factors; first, the continuing aging of native population, and second the incoming migratory fluxes from developing countries. However, most likely the first one is still predominant and, therefore, elderly people are one of the fastest-growing segments in most western societies. The incidence of meningeal pathogen infections is highly influenced by the age of the patient, and ABM is considered a disease of extreme ages of life [4].

The aim of our study was to determine the spectrum of ABM in elderly patients over the last 30 years, compared to a younger population from the same geographical area, both recruited from a community university hospital in Barcelona (Spain).

## Methods

### Study population

We used data from a large, prospective, single-hospital cohort of patients with ABM enrolled over a 30-year period at the *Hospital de la Santa Creu i Sant Pau* (Barcelona, Spain). Our institution is a 620-bed tertiary university hospital providing care for an urban area with a population of 441,392 inhabitants. From 1982 to 2010 all consecutive adults with ABM diagnosed at our Hospital were prospectively identified and followed. The study was approved by the Ethics Committee of the *Hospital de la Santa Creu I Sant Pau*.

### Diagnosis of acute bacterial meningitis

The diagnosis of ABM caused by a specific bacterial pathogen was based on the presence of consistent clinical findings (sudden onset of headache, fever, nausea, vomiting, neck stiffness and/or altered mental status) and one of the following: a positive cerebrospinal fluid (CSF) culture; or a negative CSF culture with a finding of pleocytosis and at least one of the following: a positive CSF antigen test, a positive blood culture, or identification of gram-negative diplococci on Gram stain of CSF in patients with a petechial or purpuric rash and a fulminant course. These last cases were considered to be caused by *Neisseria meningitidis*[5]. Episodes of ABM without etiologic diagnosis were included if patients had a consistent clinical picture and CSF cyto-biochemical findings consistent with purulent meningitis (pleocytosis of more than 5 polymorphonuclear leukocytes/mm^3^, hypoglycorrachia defined as a CSF/blood glucose ratio < 0.50 and high CSF protein defined as more than 45 mg/dl) despite negative blood and negative CSF Gram stain and cultures [6,7]. Patients with viral, fungal, or mycobacterial meningitis or a history of neurosurgical procedures or traumatic head/spinal injury were excluded.

From each episode, the following data were collected: sex, age, underlying conditions, co-morbidity and co-morbidity scoring according to the Charlson index, clinical characteristics, laboratory and microbiologic results (isolates and susceptibility tests), complications, treatment, and outcome. Data were collected during the index hospitalization, and each patient was seen on admission and followed after discharge by one of the authors (PD or VP).

### Microbiologic methods

Isolates were obtained from routine cultures and were identified using standard methods [8]. The disc diffusion susceptibility test was performed according to Clinical Laboratory Standards Institute (CLSI) guidelines [9], using commercially available discs (Bio-Rad™, Marnes La Coquette, France). MICs were determined using the broth micro dilution method according to the CLSI guidelines [10] using commercial panels (Sensitre,™ Trek diagnostic systems, West Sussex, England) or Etest™ (AB Biodisk, Solna, Sweden) according to the recommendations of the manufacturer. Resistance breakpoints for penicillin were MIC ≥ 0.12 mg/L for pneumococcus and meningococcus [11].

### Definitions

A subject aged > 14 years was considered an adult and when aged ≥ 65 years, elderly. Pre-admission receipt of adequate antibiotic therapy has been previously defined [12]. The interval from onset of symptoms and signs of ABM until admission was the interval symptoms-admission (ISA), whereas that from admission to the first dose of antibiotic for ABM treatment was the interval admission-therapy (IAT). Co-morbid conditions were considered to be present if the patient had a confirmed diagnosis of one or more of the following: cancer, systemic vasculitides, cirrhosis of the liver, diabetes, chronic renal failure, human immunodeficiency virus infection, heart failure, and chronic pulmonary disease [12]. Alcohol abuse was defined when alcohol intake was > 40 g daily [13]. Co-morbidity was graded according to the Charlson Co-morbidity Scale [14].

Extra-meningeal infection was considered when a patient had a focal infection distant from the Central Nervous System and the same pathogenic bacterium was isolated from the primary focus or from blood culture [15]. Appropriate antibiotic therapy was defined as administration of one or more antimicrobial agents shown to be effective against the meningeal pathogen, which achieve adequate CSF concentrations, commenced on admission or before neurological or systemic deterioration in inpatients [16]. Impaired mental status, seizures and focal neurologic signs after the first dose of antibiotics were considered neurological complications. The development of septic shock, acute respiratory failure, renal failure, and/or consumption coagulopathy was considered as extra-neurological complications if related to ABM and apparent on admission or shortly afterwards [17-22].

Nosocomial meningitis was defined as that developing more than 48 hours after admission or within one week after discharge from the hospital [23]. Coma has been defined elsewhere [17]. Inpatient deaths were considered due to ***A***BM when meningitis was the underlying and immediate cause of death. Causes of death were classified in two categories: neurological causes or systemic causes [18,23]. ABM was not considered to be the underlying cause of death if a disease process unrelated to meningitis began > 24 h after meningitis resolution and if it initiated the train of morbid events leading to death [23]. Sequelae were defined as previously outlined [12].

### Statistical analysis

Continuous variables were compared using Student’s *t*-test or the Mann–Whitney *U* test when appropriate. To compare the incidence rates and to estimate the rate ratio plus the 95% CI, we used a Poisson model. We analyzed categorical data using the *χ*^2^ test or Fisher’s exact test when indicated. Survival curves in younger patients and in those older than 65 years were compared by means of the logrank test. Statistical analyses were performed using Statistical Product and Service Solutions (SPSS) software version 16 (SPSS Inc., Chicago, IL).

## Results

### Population studied

From 1982 to 2010, 635 episodes of ABM were diagnosed in 623 adult patients, 427 in patients aged 15–64 years (Group I) and 208 in patients aged ≥ 65 years (Group II). The corresponding incidence was 4.03/100,000 and 7.40/100,000 inhabitants/year for group I and II, respectively (RR = 1.84; 95% CI: 1.56–2.17, P < 0.0001). The median yearly number of cases was 21 (95% CI: 19–25). Demographics of the population studied are summarized in Table 1. Elderly population had co-morbid conditions more frequently (Table 1). Co-morbidity predisposing to ABM was outstandingly frequent in meningitis by *Listeria monocytogenes* (87.4%) compared with other etiologies (43.4%) (RR = 1.55, 95%CI: 1.24–1.93, P = 0.0006). There were no statistically significant differences between patients from both groups in terms of gender, recurrence, and place of acquisition of ABM (Table 1).

**Table 1:** Demographics of patients with acute bacterial meningitis according to age group*

	**Group I (non-elderly)**	**Group II (elderly)**	**RR (95% CI)**	**P value**
	**N = 427**	**N = 208**		
Age (yrs)	32.0 (22.0–54.0)	74.0 (68.0–78.0)	----	< 0.0001
15–24 yrs (%)	134 (31.4)	----	----	----
25–34 yrs (%)	59 (13.8)	----	----	----
35–44 yrs. (%)	60 (14.0)	----	----	----
45–54 yrs (%)	77 (18.0)	----	----	----
55–64 yrs (%)	97 (22.7)	----	----	----
≥ 65 yrs (%)	----	208 (100)	----	----
Male (%)	205 (48.0)	99 (47.6)	0.99 (0.79–1.24)	0.9895
Co-morbidity (%)	168 (39.3)	148 (71.1)	2.49 (1.93–3.22)	< 0.0001
Cancer^† ^(%)	46 (10.8)	40 (19.2)	1.52 (1.17–1.97)	0.0051
Alcohol abuse (%)	43 (10.1)	21 (7.1)	1.00 (0.69–1.45)	0.8963
Liver cirrhosis (%)	15 (3.5)	8 (3.8)	1.06 (0.60–1.88)	0.9878
Diabetes mellitus (%)	35 (8.2)	46 (22.1)	1.94 (1.54–2.44)	< 0.0001
Chronic lung disease^‡ ^(%)	11 (2.6)	25 (12.0)	2.27 (1.27–2.91)	< 0.0001
Chronic cardiovascular disease^§ ^(%)	15 (3.5)	59 (28.6)	3.00 (2.51–3.59)	< 0.0001
Chronic renal failure (%)	17 (4.0)	21 (10.1)	1.76 (1.29–2.40)	0.0041
Chronic debilitating diseases^¶ ^(%)	8 (1.9)	17 (8.2)	2.17 (1.62–2.91)	0.0003
Immunosuppression^ll ^(%)	60 (14.0)	42 (20.2)	1.36 (1.04–1.77)	0.0428
Charlson Index ≥ 1	134 (31.4)	136 (65.4)	2.55 (2.00–3.24)	< 0.0001
0 (%)	283 (68.6)	72 (34.6)	0.42 (0.33–0.53)	< 0.0001
1–2 (%)	87 (20.4)	80 (38.5)	1.75 (1.41–2.17)	< 0.0001
3–4 (%)	22 (5.1)	42 (20.2)	2.26 (1.81–2.81)	< 0.0001
≥ 5 (%)	25 (5.8)	14 (5.7)	1.10 (0.71–1.70)	0.7984
Nosocomial acquisition (%)	14 (3.3)	11 (5.3)	1.36 (0.86–2.15)	0.3149
Recurrent meningitis (%)	18 (4.2)	10 (4.8)	1.09 (0.66–1.82)	0.8924

Values expressed as median and interquartile range unless otherwise specified.*Patients may have more than a co-morbid condition. ^†^ includes: Solid (53): brain (9), lung (6), breast (6), cavum (6), prostate (4), colon (3), endometrium (2), pharynx (2), ovarian (2), urinary bladder (2), pleural mesothelioma (2), skin (2), testes (1), endometrial (1), cervix (1), sphenoidal sinus (1), oesophagus (1), gastric (1), and vagina (1), and haematological (33) cancers: multiple myeloma (8), Hodgkin’s lymphoma (3), non-Hodgkin’s lymphoma (6), acute leukemias (8), chronic leukemias (7), and Waldenström disease (1). ^‡ ^Includes: chronic bronchitis (28), asthma (4), idiopathic pulmonary fibrosis (2), other pneumopathies (2) ^§ ^Includes: arterial hypertension (40), coronary heart disease (18), congestive heart failure (15), valvular disease (8), chronic arrythmias (3), and Buerguer ‘s disease (1). ^¶^Include: prior stroke (9), senile dementia (7), hyperthyroidism (3), hypothyroidism (2), cerebral palsy (2), chronic hydrocephalus (2), amyloidosis (1). ^ll^Includes^:^ antineoplastic chemotherapy (49), hypogammaglobulinemia (22), chronic corticosteroid therapy (21), AIDS (6), splenectomy (6), hypocomplementemia (5), neutropenia (4), and radiotherapy (4).

### Clinical features

The clinical features of patients with ABM are summarized in Table 2. Patients from group II had a higher probability, although not statistically different, of having received out-of-hospital antibiotic therapy. An extra-meningeal focus of infection was found more frequently in patients from group II, and this was especially true for pneumonia and infra-diaphragmatic foci of infection (RR = 1.66; 95% CI: 1.20–2.29, P = 0.0126). Elderly patients more frequently lacked fever (although the difference did not reach statistical significance), neck stiffness and skin rash, but had an altered level of consciousness more often (Table 2). Despite being brought to the hospital with an interval similar to that of younger patients, elderly patients suffered a greater delay from arrival to the hospital to starting antibiotic therapy. Cerebral computed tomography (CT) on admission was performed in 163 (57.8%), and 113 (75.8%) patients from groups I and II, respectively (Table 2).

**Table 2:** Clinical features of patients with acute bacterial meningitis according to age group*

	**Group I (non-elderly)**	**Group II (elderly)**	**RR (95% CI)**	**P value**
	**N = 427**	**N = 208**		
Interval symptoms-admission (hrs)	24.0 (24.0–37.0)	27.5 (24.0–37.0)	----	0.3822
Prior antimicrobial therapy (%)	126 (29.5)	77 (37.0)	1.25 (1.00–1.57)	0.0696
Extrameningeal infection^† ^(%)	96 (22.5)	79 (37.9)	1.61(1.29–2.00)	< 0.0001
Pneumonia (%)	17 (4.0)	20 (9.6)	1.72 (1.25–2.37)	0.0077
ENT infection (%)	56 (8.8)	33 (5.2)	1.16 (0.86–1.56)	0.4149
Urinary tract infection (%)	9 (1.4)	18 (2.8)	2.13 (1.59–2.86)	0.0003
Fever (%)	412 (96.5)	193 (92.8)	0.64 (0.44–0.93)	0.0625
Level of consciousness (%)				
Normal (%)	174 (40.7)	49 (23.6)	0.57 (0.43–0.75)	< 0.0001
Abnormal (%)	229 (62.6)	142 (68.3)	1.53 (1.20–1.93)	0.0006
Coma (%)	24 (5.6)	17 (8.2)	1.39 (0.95–2.04)	0.1639
Neck stiffness (%)	357 (83.6)	129 (62.0)	0.50 (0.41–0.62)	< 0.0001
Meningeal triad present (%)	208 (48.7)	104 (50.0)	1.04 (0.83–1.29)	0.8275
Skin rash (%)	153 (35.8)	19 (9.1)	0.27 (0.17–0.62)	< 0.0001
Interval admission-therapy (hrs)	3.0 (1.0–6.0)	4.0 (2.0–12.0)	----	< 0.0001
Focal neurologic signs on admission (%)	63 (14.7)	43 (20.7)	1.30 (1.00–1.69)	0.0777
Seizures on admission (%)	33 (7.7)	13 (6.2)	0.85 (0.53–1.37)	0.6091
Cerebral computed tomography (%)	163 (57.8)	113 (75.8)	1.55 (1.24–1.93)	0.0002

Values expressed as median and interquartile range unless otherwise specified. ENT = ear, nose and throat. *A patient may have more than an extrameningeal focus of infection. ^† ^Includes: otiitis (73), pneumonia (37), urinary tract infection (27), paranasal sinusitis (23), endocarditis (7), pyelonephritis (4), acute bronchitis (4), tonsillitis (3), parappharyngeal abscess (2), cholecystitis (2), peritonitis (2), ventriculo-peritoneal shunt (2), dental abscess (2), scrotal abscess (1), psoas abscess (1), i.v. catheter (1), pacemaker electrode infection (1).

### CSF findings, microbiologic features and etiology

CSF cyto-biochemical and microbiological findings are summarized in Table 3. CSF protein content was significantly higher in patients from group II whereas they had a lower diagnostic yield for the CSF Gram-stained smear. The median CSF/blood glucose ratio was not statistically different for immunosuppressed and immunocompetent patients after excluding diabetic patients (0.240 {Interquartile range: 0.091–0.405} vs. 0.220 {IQR: 0.0070–0.440}, P = 0.5402). The median CSF cell count in immunosuppressed patients was 560 cells/mm^3^ (IQR: 173–1891) whereas in immunocompetent patients it was 1178 (IQR: 327–3036) cells/mm^3^ (P = 0.0029). Blood cultures were most frequently positive in elderly patients, although the difference did not reach statistical significance. Meningococcal meningitis was diagnosed less frequently whereas pneumococcal meningitis was diagnosed more often in the elderly, although the former difference was not statistically significant. Listerial meningitis, gram-negative bacillary meningitis, and meningitis of unknown origin also showed increases in group II (Table 3). Meningococcal isolates from elderly patients showed decreased susceptibility to penicillin more frequently (27.3% vs. 5.5%, RR = 1.57; 95% CI: 0.99–2.47, P = 0.0025), whereas penicillin-resistant pneumococcal isolates were evenly distributed between both groups (14.3% vs. 11.9%, RR = 1.09; 95% CI: 0.41–1.69, P = 0.8771). The percentage of serogroup C meningococcal isolates fell from 45.8% to 11.1% before and after the vaccination campaign of the year 2000 (RR = 0.25; 95% CI: 0.08–0.75 P = 0.0050). Similarly, pneumococcal serotypes included in the heptavalent vaccine fell from 38.4% to 22% after 2001 (OR = 2.21; 95% CI: 0.99–5.09, P = 0.0539).

**Table 3:** CSF findings, microbiologic features and etiology of acute bacterial meningitis according to age group

	**Group I (non-elderly)**	**Group II (elderly)**	**RR (95% CI)**	**P value**
	**N = 427**	**N = 208**		
**CSF findings**				
Protein content, g/l	3.2 (1.5–6.5)	3.8 (1.8–7.3)	----	0.0306
mg/dl	320 (150–650)	380 (180–730)		
CSF/plasma glucose ratio	0.24 (0.07–0.45)	0.22 (0.08–0.42)	----	0.7681
Cells/mm^3^	1022 (301–2993)	1026 (290–2968)	----	0.7490
Predominance PML ^†^	385 (94.4)	183 (91.5)	0.76 (0.52–1.11)	0.2446
Positive CSF Gram stained smear (%)	199 (46.6)	68 (32.7)	0.67 (0.52–0.85)	0.0012
Positive CSF culture (%)	309 (72.4)	141 (67.8)	0.87 (0.68–1.09)	0.2721
Positive blood culture (%)^‡^	184 (43.4)	104 (51.5)	1.25 (1.00–1.58)	0.0616
**Etiology **^§^				
Meningococcal (%)	179 (41.9)	24 (11.5)	0.28 (0.19–0.41)	< 0.0001
Pneumococcal (%)	91 (21.3)	59 (28.4)	1.28 (1.01–1.63)	0.0622
Listerial & grampositive bacilli (%)	33 (7.7)	29 (13.9)	1.93 (1.12–2.01)	0.0196
Other Gram-positive cocci (%)	22 (5.1)	13 (6.2)	1.14 (0.73–1.79)	0.7012
Gram-negative bacilli (%)	18 (4.2)	21 (10.1)	1.72 (1.25–2.35)	0.0065
*Haemophilus influenzae *(%)	9 (2.1)	5 (2.4)	1.09 (0.54–2.23)	0.9685
Mixed (%)	2 (0.5)	2 (1)	1.53 (0.57–4.11)	0.8393
Other	2 (0.5)	1 (0.5)	1.02 (0.20–5.06)	0.5517
Unknown origin (%)	71 (16.5)	54 (25.9)	1.43 (1.12–1.82)	0.0076

Values expressed as median and interquartile range unless otherwise specified. CSF = cerebrospinal fluid. PML polymorphonuclear leukocytes. ^†^ Differential count performed in 608 patiens. ^‡^Blood culture performed in 624 patients. ^§ ^Gram-positive bacilli: *Listeria monocytogenes* (60) and *Bacillus cereus *(2). Gram-positive cocci: *Staphylococcus aureus* (12), *Streptococcus agalactiae *(8), *Streptococcus viridans* (4), *Streptococcus pyogenes *(3), *Enterococcus faecalis *(2), *Staphylococcus epidermidis *(2), *Streptococcus anginosus *(1), *Staphylococcus sp*. (1), Group G streptococcus (1), *Streptococcus bovis* (1). Gram-negative bacilli: *Escherichia coli *(17), *Pseudomonas aeruginosa* (8), *Pseudomonas sp*. (3), *Serratia sp*. (2), *Klebsiella sp*. (2), *Proteus mirabilis* (2), *Serratia marcescens *(1), *Acinetobacter baumanii* (1), *Bacteroides melaninogenicus *(1), and *Citrobacter freundii* (1), and *Enterobacter cloacae* (1). Mixed meningitis: *Bacteroides intermedius* + *Streptococcus viridans *(1), *Enterobacter cloacae* + *Streptococcus viridans *(1), *Peptostreptococcus sp*. + anaerobic unidentified GNB (1), and *Peptostreptococcus sp*. + *Bacteroides sp*. (1). Other: *Brucella sp*. (2) and *Neisseria subflava *(1).

### Evolving features and outcome

Elderly patients developed more frequently coma, and cranial nerve palsies of new appearance. Patients from group II also developed acute respiratory failure and acute renal failure more frequently (Table 4). Almost 90% of patients in each group received adequate empiric antibiotic therapy.

**Table 4:** Evolving features and outcome of acute bacterial meningitis according to age group*

	**Group I (non-elderly)**	**Group II (elderly)**	**RR (95% CI)**	**P value**
	**N = 427**	**N = 208**		
**Neurologic complications (%)**	**80 (18.7)**	**64 (30.8)**	**1.52 (1.21–1.90)**	**0.0009**
New focal neurologic signs (%)	23 (5.4)	16 (7.7)	1.27 (0.86–1.89)	0.3372
Coma (%)	57 (13.3)	48 (23.1)	1.51 (1.18–1.94)	0.0028
Seizures (%)	46 (10.7)	33 (15.9)	1.33 (1.00–1.77)	0.0897
New cranial palsies (%)	17 (4.0)	17 (8.2)	1.57 (1.10–2.25)	0.0439
**Extra-neurologic complications (%)**	**109 (25.5)**	**94 (45.2)**	**1.75 (1.41–2.18)**	**< 0.0001**
Septic shock (%)	65 (15.2)	35 (16.8)	1.08 (0.80–1.44)	0.7125
Acute respiratory failure (%)	60 (14.0)	46 (22.1)	1.42 (1.10–1.82)	0.0145
Acute renal failure (%)	40 (9.4)	48 (23.1)	1.86 (1.48–2.35)	< 0.0001
Consumption coagulopathy (%)	39 (9.1)	15 (7.2)	0.84 (0.54–1.31)	0.5071
**Therapeutics**				
Adequate empiric antibiotic therapy (%)	398 (93.2)	187 (89.9)	0.76 (0.54–1.08)	0.1956
Dexamethasone therapy (%)	142 (33.2)	83 (39.9)	1.21 (0.97–1.52)	0.1198
Vasoactive drugs (%)	69 (16.1)	38 (18.3)	1.10 (0.83–1.46)	0.5797
Mechanical ventilation (%)	67 (15.7)	39 (18.7)	1.15 (0.87–1.52)	0.3915
Dialysis (%)	9 (2.1)	8 (3.8)	1.45 (0.87–2.44)	0.3116
**Outcome**				
Overall mortality rate (%)	50 (11.7)	62 (29.8)	1.98 (1.60–2.46)	< 0.0001
Meningococcal (%)	8 (4.5)	3 (12.5)	2.49 (0.88–7.10)	0.2494
Pneumococcal (%)	14 (15.0)	19 (31.7)	1.68 (1.14–2.48)	0.0259
Listerial & grampositive bacilli (%)	7 (21.2)	9 (31.0)	1.29 (0.75–2.23)	0.5544
Other grampositive cocci (%)	9 (40.9)	6 (46.1)	1.14 (0.48–2.70)	0.9597
Gramnegative bacilli (%)	10 (55.5)	12 (57.1)	1.03 (0.57–1.86)	0.8225
*Haemophilus influenzae *(%)	1 (11.1)	0 (0.0)	1.50 (0.30–7.43)	0.7320
Mixed (%)	0 (0.0)	0 (0.0)	----	----
Other	0 (0)	0 (0.0)	----	----
Unknown etiology (%)	1 (1.4)	13 (24.1)	2.51 (1.89–3.34)	0.0002
Death attributable to meningitis (%)	42 (9.8)	43 (20.7)	1.67 (1.30–2.13)	0.0004
Post-meningitic sequelae^† ^(%)	42 (11.1)	21 (14.4)	1.22 (0.83–1.78)	0.3945

Values expressed as median and interquartile range unless otherwise specified. *A patient may have more than one complication or sequelae. ^† ^Sequelae: total or partial deafness (19), limb palsy (18), cranial nerve palsy (13), memory disturbances (10), vegetative state (5), speech disturbances (4), polyneuritis (3), blindness (3), bilateral leg amputation (2), seizures (2), myoclonia (2), hydrocephalus (1), chronic leg ulcers (1), long-term tracheostomy (1), and chronic renal failure (1).

**Figure 1:**
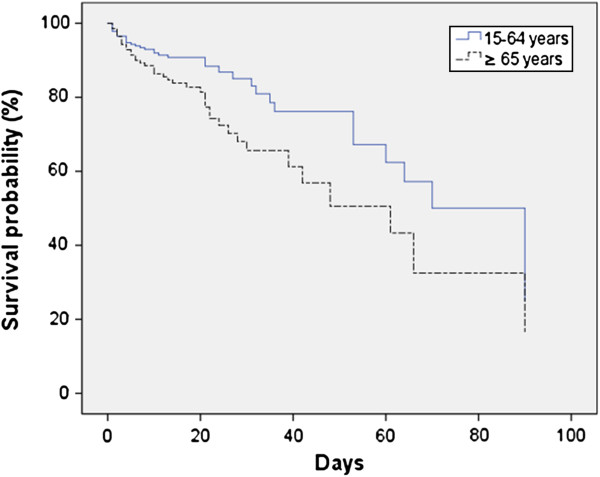
Survival curves for adult patients with acute bacterial meningitis according to age (P = 0.007).

The overall mortality ratio was significantly higher for patients in group II (29.8% vs. 11.7%) (Table 4). This higher mortality rate was caused by a higher mortality rate of pneumococcal and of meningitis of unknown origin. Survival curves for both groups of patients showed an earlier probability of death for elderly patients (Figure 1). When death directly attributable to ABM was considered, the probability of dying was again higher for elderly patients. Development of complications conveyed higher rates of mortality in both groups, without statistically significant differences between them (Table 5). There were no differences between groups with respect to post-meningitic sequelae.

**Table 5:** Outcome of acute bacterial meningitis according to age group and severity indexes*

	**Group I (non-elderly)**	**Group II (elderly)**	**RR (95% CI)**	**P value**
	**N = 427**	**N = 208**		
**Type of complication (%)**				
None (%)	3/292 (1.03)	3/93 (3.2)	3.07 (0.63–14.97)	0.3263
Neurologic complications (%)	9/26 (34.6)	11/21 (52.4)	1.34 (0.64–2.80)	0.6124
Extra-neurologic complications (%)	8/55 (14.5)	11/51 (29.4)	1.79 (0.82–3.93)	0.2085
Both types (%)	30/54 (55.5)	33/43 (76.7)	1.22 (0.83–1.79)	0.4041
**Extra-neurologic complications (%)**				
New focal neurologic signs (%)	13/23 (56.5)	11/16 (68.7)	1.13 (0.60–2.12)	0.9105
Coma (%)	37/57 (64.9)	41/48 (85.4)	1.17 (0.84–1.64)	0.4429
Seizures (%)	16/46 (34.8)	21/33 (63.6)	1.51 (0.88–2.58)	0.1908
New cranial palsies (%)	4/17 (23.5)	11/17 (64.7)	2.06 (0.76–5.58)	0.2270
**Extra-neurologic complications (%)**				
Septic shock (%)	30/65 (46.1)	23/35 (74.3)	1.35 (0.89–2.04)	0.2178
Acute respiratory failure (%)	22/60 (36.7)	27/46 (58.7)	1.38 (0.86–2.20)	0.2360
Acute renal failure (%)	19/40 (47.5)	27/48 (56.2)	1.12 (0.69–1.80)	0.7823
Consumption coagulopathy (%)	19/39 (48.7)	11/15 (73.3)	1.29 (0.72–2.31)	0.5497

Values expressed as patients who died/total number of patients with complication. *A patient may have more than one complication or sequelae.

## Discussion

Our study shows that elderly patients are at much higher risk of developing ABM than younger adults. We also observed a change in the etiology of the disease, *Neisseria meningitidis* ranked first for younger patients, whereas it was surpassed by pneumococcal, listerial, and meningitis of unknown origin in the elderly. Another important, although expectable, change is the increased prevalence of co-morbid conditions among elderly people. This may have an impact, not only on the etiology of ABM, but also on the rate of complications and eventually on mortality. Other differing findings for elderly people include a higher chance of having received out-of-hospital antibiotic therapy, more frequently lacking fever, neck stiffness and skin rash, developing neurologic and extra-neurologic complications more frequently, and having an increased risk of death because of ABM. In summary, the linkage and interaction of higher co-morbidity, different etiologic spectrum, more pre-admission antibiotic therapy and subtler clinical manifestations may contribute to explain most of the differences between elderly and younger patients.

However, our work has inherent limitations. First, our study is based on a single hospital and this may imply that the results may only be applicable to places with a population structure and local ecology similar to ours. However, changes in the spectrum of ABM similar to those observed in our study have been described, especially those referring to the epidemiology of meningococcal disease [2,24-26]. Moreover, the pattern of population change documented in Spain, has also been reported in other Western, mainly European countries [27]. Therefore, the population scenario may resemble that described in the present work. Our study spans over a long period of time; during 30 years it is obvious that the treatment and management of critically ill patients and of ABM have changed a lot. Although the case record forms were standardized and remained the same throughout the study, they may reflect differing sensitivities among treating physicians and in the end are a reflection of the “real world”. An important part of this real world is meningitis of unknown origin which is the most diagnosis-challenging ABM. We had strict criteria for this diagnosis, and when reasonable doubts arose, the case was not included in our series.

Changes in population structure contribute to changes in the spectrum of ABM. During the study period, Barcelona population has ranged from 1,754,900 inhabitants in 1982 to 1,619,337 in 2010, with a minimum of 1,508,805 in 1996 [28]. However, the elderly population increased from 252,415 inhabitants in 1986 to 419,988 in 2006, whereas the population aged 15–24 years decreased from 266,791 in 1986 to 157,737 in 2006 [28]. Therefore, a decrease in the population more prone to meningococcal meningitis was observed together with an increase in the population more susceptible to having pneumococcal and listerial meningitis. Surprisingly, meningitis of unknown origin also significantly increased in group II, which may be explained by a higher prevalence of out-of-hospital antibiotic therapy among elderly people. The negative impact of pre-admission antibiotic therapy on the positivity of microbiological tests is well known [12,29].

Although *N*. *meninigitidis* is not a common cause of ABM in elderly patients, our epidemiological environment has for many years been one of high endemicity of meningococcal disease [24,30,31]. Moreover, the incidence of disease due to serogroup C meningococci increased in Spain from the mid-nineties [32]. This change of pattern led to the carrying out of a mass vaccination campaign against serogroup C in Spain and to inclusion of the meningococcal C conjugate vaccine in the routine vaccination schedule [33]. Since group C meningococci have a predilection for causing disease in older adults [12], the vaccination campaign may also have contributed to the decreased incidence of meningococcal meningitis among the elderly as our results show. This can be explained by the increase in herd immunity [34].

With respect to clinical findings, although elderly patients were brought to the hospital not later than younger ones, the time before first antibiotic dose was significantly longer. There are a cluster of circumstances that may explain this finding; elderly patients had taken out-of- hospital antibiotic therapy more frequently, and this, together with being older, makes the symptoms and signs of ABM subtler, as exemplified by the less frequent presence of symptoms and/or signs of ABM and skin lesions [15,29,35-38], which may eventually lead to a diagnostic delay. But, above all, elderly patients had a cerebral CT performed more frequently, and this delayed the spinal tap and consequently the start of antibiotic therapy. Delay in starting antibiotic therapy has been advocated as a main factor in ABM-driven mortality [36-42]. Therefore, a high index of suspicion of ABM is needed in elderly patients with fever and CNS dysfunction to rule out meningeal infection eventually leading to a prompt spinal tap and early starting of empiric antibiotic therapy.

There were no differences between both groups in terms of CSF parameters except for a higher CSF protein content for group II patients, which is most likely due to the predominance of pneumococcal, listerial and gram-negative bacillary meningitis in this group. We know that these meningitis have a higher CSF protein content than meningococcal which predominates among group I patients (data not shown). Underlying immune suppression may cause changes in CSF parameters; and we found like Erdem et al. [37] lower CSF counts in immunosuppressed patients but not a different CSF/blood glucose ratio. In addition, the diagnostic yield of CSF Gram-stained smear was inferior in elderly patients, but we must take into account that pre-admission antibiotic therapy had been taken by 37% of patients, and we know that this is the most frequent cause of meningitis with negative CSF Gram-stained smears [35,43,44].

Both neurologic and extra-neurologic complications appeared more frequently in elderly patients. This may be explained by two facts; first, the physiological reserve and the ability of many organs and systems to respond to insults decrease with age, and second, in elderly patients, the baseline organ function may be compromised because of prior co-morbid conditions. Therefore, ABM and/or its associated systemic consequences may further damage an already dysfunctional organ, eventually leading to its failure. However, once present, they usually convey high mortality rates both in young and elder patients. The excess mortality of ABM in elderly people is a usual finding in related literature [37,38,44,45]. In fact, this difference in mortality rate is mainly driven by an excess mortality in pneumococcal and meningitis of unknown origin.

## Conclusions

In summary, elderly people are at higher risk of having ABM than younger adults, ABM appears more frequently in patients with co-morbid conditions, is associated with a longer interval from admission to antibiotic therapy, is caused by *S*. *pneumoniae*, and patients develop neurologic and extra-neurologic complications more frequently. ABM in elderly patients is associated with an earlier and higher mortality rate than in younger ones.

## Competing interests

There are no competing interests to be disclosed.

## Authors’ contributions

PD and VP designed the database, analyzed the results and wrote the paper. NB analyzed the results and performed statistical studies. PC performed the microbiological studies and wrote the microbiological parts of the study. All authors have read and approved the final manuscript.

## Pre-publication history

The pre-publication history for this paper can be accessed here:

http://www.biomedcentral.com/1471-2334/13/108/prepub
